# Clinical Relevance and Immunosuppressive Pattern of Circulating and Infiltrating Subsets of Myeloid-Derived Suppressor Cells (MDSCs) in Epithelial Ovarian Cancer

**DOI:** 10.3389/fimmu.2019.00691

**Published:** 2019-04-03

**Authors:** Karolina Okła, Arkadiusz Czerwonka, Anna Wawruszak, Marcin Bobiński, Monika Bilska, Rafał Tarkowski, Wiesława Bednarek, Iwona Wertel, Jan Kotarski

**Affiliations:** ^1^The First Department of Gynecologic Oncology and Gynecology, Medical University of Lublin, Lublin, Poland; ^2^Tumor Immunology Laboratory, Medical University of Lublin, Lublin, Poland; ^3^Department of Virology and Immunology, Maria Curie-Skłodowska University, Lublin, Poland; ^4^Department of Biochemistry and Molecular Biology, Medical University of Lublin, Lublin, Poland

**Keywords:** epithelial ovarian cancer, myeloid-derived suppressor cells, immunosuppression, prognosis, liquid biopsy

## Abstract

Myeloid-derived suppressor cells (MDSCs) expansion is a hallmark of cancer. Three major MDSC subsets defined as monocytic (M)-MDSCs, polymorphonuclear (PMN)-MDSCs and early stage (e)MDSCs can be revealed in human diseases. However, the clinical relevance and immunosupressive pattern of these cells in epithelial ovarian cancer (EOC) are unknown. Therefore, we performed a comprehensive analysis of each MDSC subset and immunosupressive factors in the peripheral blood (PB), peritoneal fluid (PF), and the tumor tissue (TT) samples from EOC and integrated this data with the patients' clinicopathological characteristic. MDSCs were analyzed using multicolor flow cytometry. Immunosuppressive factors analysis was performed with ELISA and qRT-PCR. The level of M-MDSCs in the PB/PF/TT of EOC was significantly higher than in healthy donors (HD); frequency of PMN-MDSCs was significantly greater in the TT than in the PB/PF and HD; while the level of eMDSCs was greater in the PB compared with the PF and HD. Elevated abundance of tumor-infiltrating M-MDSCs was associated with advanced stage and high grade of EOC. An analysis of immunosuppressive pattern showed significantly increased blood-circulating ARG/IDO/IL-10-expressing M- and PMN-MDSCs in the EOC patients compared with HD and differences in the accumulation of these subsets in the three tumor immune microenvironments (TIME). This accumulation was positively correlated with levels of TGF-β and ARG1 in the plasma and PF. Low level of blood-circulating and tumor-infiltrating M-MDSCs, but neither PMN-MDSCs nor eMDSCs was strongly associated with prolonged survival in ovarian cancer patients. Our results highlight M-MDSCs as the subset with potential the highest clinical significance.

## Introduction

According to GLOBOCAN 2018 report, 295,414 women will be newly diagnosed with ovarian cancer, resulting in 184,799 deaths worldwide in 2018 and it is estimated that it can be above 400,000 incidence cases and near 300,000 of deaths in 2040 (1). The highest mortality rate of epithelial ovarian cancer (EOC) among gynecological tumors has not changed significantly for decades. Although initial response to cytoreductive surgery and aggressive regimens of chemotherapy are often excellent, and have led to increased survival rates of patients with advanced tumor, nearly 90% will develop relapse disease that is resistant to chemotherapy (2, 3), possibly due to tumor-favorable switch of immune cells. In the light of our failure to improve long-term survival of patients and deficient screening algorithms there is an urgent need to address the key cellular/molecular components by which tumor progression and poor clinical outcome occur.

Cancers are not just simple masses of tumor cells but complex/dynamic/spatially heterogeneous “rogue” arena where malignant cells and non-malignant/immune cells interact to create tumor milieu. Although non-malignant cells can comprise >50% of the mass of primary/metastatic tumors, there are still many open questions regarding their clinical relevance (4). In the tumor immune microenvironment (TIME), the principal “mission” of immune cells is to execute an antitumor program, but a portion of such cells becomes a confederate of the tumor (5–7). Amidst this complexity, myeloid-derived suppressor cells (MDSCs) can play a pivotal role.

We have previously observed that EOC is a profoundly immunosuppressive disease that hampers the efficacy of (immuno)therapy. Even though we and others have characterized the role of immune cells in the TIME in the modulation of tumor growth/metastasis/drug resistance as well as their clinical impact (8–13), to date the myeloid component, particularly tumor-promoting, and immunosuppressive MDSCs and the clinical significance of these cells during EOC progression has not been fully elucidated.

MDSCs are a pivotal immunosuppressive and tumor-promoting heterogenous cell population in the TIME of tumor-bearing mice and cancer patients (14). Originally, MDSCs were found in tumor-bearing mice, where two major subsets exist, including CD11b+Ly6G^−^Ly6C^high^ M-MDSCs and CD11b^+^Ly6G^+^Ly6C^low^ PMN-MDSCs (15, 16). Lack of specific markers for human MDSCs hamper clinical analysis. In view of the frequent discrepancies, researchers established immunophenotype criteria to define MDSCs and distinguished three subsets of this population including: monocytic (M)-MDSCs; HLA-DR^−/low^CD11b^+^CD14^+^CD15^−^, polymorphonuclear (PMN)-MDSCs; HLA-DR^−/low^CD11b^+^CD14^−^CD15^+^ and early-stage eMDSCs; HLA-DR^−/low^CD11b^+^Lin^−^CD33^+^ (17). MDSCs use vast inflammatory mediators to suppress anti-tumor immunity, e.g., arginase 1 (ARG1), transforming growth factor β (TGF-β), interleukin 10 (IL-10), and indoleamine 2,3-dioxygenase (IDO) (8, 14). Since the mid-1990s a growing body of evidence has shed considerable light on the important roles of myeloid cells in tumor growth/metastasis/course of cancer disease, however more inquiring studies from a clinical standpoint are needed (18–20).

Our previous findings have identified for the first time the clinical impact of tumor-infiltrating CD33^+^ MDSCs in high-grade serous EOC. We found that tumor-infiltrating CD33^+^ MDSCs were significantly associated with shorter overall survival (OS) and a reduced disease-free interval. Interestingly, our data revealed that MDSCs promote stemness of cancer cells by inducing microRNA101 and suppressing the corepressor C-Terminal Binding Protein 2 (CtBP2) (21). Obermajer's group showed that CD11b^+^CD14^+^CD33^+^ MDSCs from ascites are recruited into the TIME of EOC through the CXCL12-CXCR4 pathway (22). Wu's group revealed that IL-6/IL-10 from ascites synergistically expand CD14^+^HLA-DR^−/low^ MDSCs in EOC patients and high abundance of ascites/blood-derived MDSCs was associated with poor prognosis (23). Recently, Santegoets's group has demonstrated that blood M-MDSC/dendritic cell ratio is an independent predictive factor for EOC survival (24). Besides, a meta-analysis of 40 studies comprising 2,721 patients revealed that a higher preatreatment cMDSCs level is a potential prognostic parameter in patients with solid cancer (25). Interestingly, the concept of peripheral immunoscore (analysis of immune cells including MDSCs within PBMC) has been proposed as a predictive baseline biomarker in two patients cohorts receiving cancer vaccines. The peripheral immunoscore of refined subsets demonstrated significant differences in PFS for breast cancer patients (docetaxel plus vaccine treatment), and in prostate cancer patients (radionuclide plus vaccine treatment) (26). To date, the Food and Drug Administration (FDA) has not approved measurement of any circulating immunological biomarkers (i.e., immune cells, proteins, genes, miRNAs) for patients with cancer. However, reliability, low-cost, multiplexing capacities, sensitivity, short analysis time, user-friendly, flexibility biomarkers from peripheral blood [liquid biopsy (18)] would be ideal to provide clinical guidance in routine clinical practice.

Notwithstanding, to date, nobody assessed the clinical relevance and immune pattern of three different subpopulations of MDSCs in the three TME of ovarian cancer patients.

Guided by all prior reports concerning the detrimental role of MDSCs in malignancies, immunosuppression in EOC milieu, together with the clinical value of MDSCs, we focused on miscellaneous characteristic of MDSCs. Here, we performed a comprehensive analysis of the frequency, phenotype and immunosupressive pattern of three MDSC subsets (i.e., M-/PMN-/eMDSCs) and related immunosuppressive factors in the three EOC compartments (i.e., PB/PF/TT) and integrated this data with patients' clinical characteristic.

## Materials and Methods

### Study Population

The ethics committee of the Medical University of Lublin approved this study. All patients/HD signed an informed consent. Participants were recruited at The First Department of Oncological Gynecology and Gynecology (Clinical Hospital No. 1, Poland), from 2009 until 2016. We obtained PB (*n* = 47), PF (*n* = 29), and TT (*n* = 32) from EOC patients. Exclusion criteria for study included serious intercurrent chronic/acute illnesses, the presence of infections/autoimmune disorders/allergic, concurrent second malignancy other than EOC, previous chemotherapy/radiation therapy prior to surgery, patients on immunosuppressive agents. Inclusion criteria were: at least 18 years of age and histologically confirmed diagnosis of EOC. PB samples were also collected from sex-/age-matched HD (*n* = 15) as control. Characteristics of patients/HD are reported in Supplementary Table 1.

### Cell Isolation

Venous blood samples were collected before the surgical procedure. PF/TT were collected aseptically during the operation. Plasma/PF samples were rendered cell-free by centrifugation (1,500 rpm/10 min) and the supernatants were stored at −80°C for later analysis. Mononuclear cells (MCs) from PB/PF were isolated by density gradient centrifugation on Lymphoprep™ (Stemcell Technologies, Canada). For isolation of tumor-infiltrating MCs, freshly resected TT was minced, placed into a gentleMACS C tube and processed using Tumor Dissociation Kit (MiltenyiBiotec). The resulting cell suspension was filtered through 70-mm mesh filter (BD Biosciences) and subjected to the density centrifugation as described above. MCs were isolated within 2 h of draw, cryopreserved and stored in liquid nitrogen until use.

### Flow Cytometry

**Figure 1:**
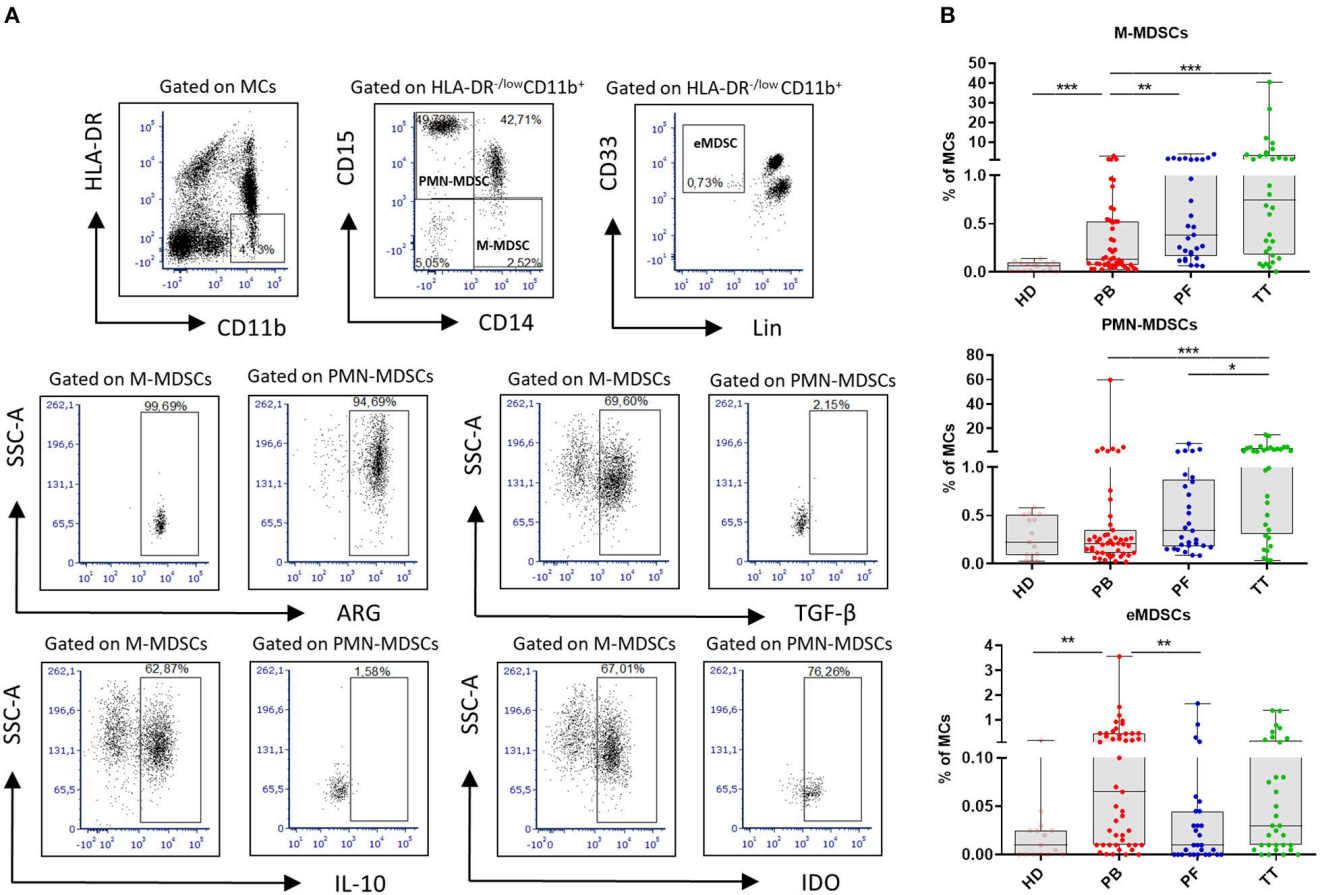
Analysis of myeloid-derived suppressor cells (MDSCs) in EOC. Mononuclear cells (MCs) obtained from peripheral blood (PB) (*n* = 47), peritoneal fluid (PF) (*n* = 29), and tumor tissue (TT) (*n* = 32) from epithelial ovarian cancer (EOC) patients and healthy donors (HD) (*n* = 15) were assessed by multicolor flow cytometry. MCs were stained for MDSCs using fluorochrome-labeled antibodies against HLA-DR, CD11b, CD14, CD15, CD33, Lin, arginase 1 (ARG1), indolamine 2,3-dioxygenase (IDO), interleukin 10 (IL-10), and transforming growth factor β (TGF-β). A morphological gate including mononuclear cells (based on SSC and SFC properties) and exclusion of doublets (based on FSC-A vs. FSC-H parameters) were applied before gating for MDSC subsets. Names above cytometric plots indicate the population gated that was analyzed. Markers analyzed are indicated in the axis of each cytometric plot. Representative dot plots of three subsest of MDSCs, including monocytic (M)-MDSCs (HLA-DR^−/low^CD11b^+^CD14^+^CD15^−^ cells), polymorphonuclear (PMN)-MDSCs (HLA-DR^−/low^CD11b^+^CD14^−^CD15^+^ cells) and early-stage eMDSCs (HLA-DR^−/low^CD11b^+^Lin^−^CD33^+^ cells) identified using a gating strategy are shown **(A)**. The frequencies of M-, PMN- and eMDSCs in the PB/PF/TT and healthy donors (HD) are presented as the percentage of the MCs **(B)**. The frequencies of ARG^+^/IDO^+^/IL-10^+^/TGF-β^+^ M-, PMN-MDSCs are shown as the percentage within the total respective subset. Each point corresponds to an individual patient or a HD. Boxes indicate the 25 to 75th percentiles. The horizontal lines within the boxes are the median values and the whiskers indicate the minimum and maximum values. Asterisks represent statistical significance (^*^p < 0.05; ^**^p < 0.01; ^***^p < 0.001;).

For MDSCs characterization the following mAbs were used: anti-CD14/Pe-Cy7, anti-CD11b/APC-Cy7, anti-HLA-DR/PerCP-Cy5.5, anti-Lineage cocktail 1/FITC (BD Biosciences), anti-CD33/PE, anti-CD15/APC, anti-TGF-β/FITC (BioLegend) and anti-ARG1/AlexaFluor488, anti-IDO/AlexaFluor488, anti-IL-10/FITC (R&D Systems). For intracellular staining, the cells were fixed/permeabilized using a Cytofix/Cytoperm Fixation/Permeabilisation Kit (BD Biosciences, USA). Nonspecific staining was prevented by using FcR Blocker (Miltenyi Biotec, Bergisch Gladbach, Germany). Cells were analyzed with BD FACSCanto flow cytometer using FACS DIVA software (BD Biosciences, USA) and FCS express (*De Novo* Software, Los Angeles, CA). The gating strategy for MDSCs is shown in Figure 1A.

### ELISA

IDO/IL-10/TGF-β in the plasma/PF were analyzed using ELISA kits (Wuhan EIAab Science; IDO analysis, and Affymetrix eBioscience; IL-10/TGF-β analysis). Proteins were quantified with ELX-800 Universal Microplate Reader (Bio-Tek, Winooski, VT) using Gen5™ software (Bio-Tek, Instruments, USA).

### Arginase Activity Assay

ARG1 activity was assessed with QuantiChrom™ Arginase Assay Kit (DARG-100) (BioAssay Systems) using Infinite M200 Pro microplate reader (Tecan, Männedorf, Switzerland).

### RNA Preparation and qRT-PCR

Total RNA was extracted from PB/PF/TT MCs OC patients (*n* = 8) using AllPrep DNA/RNA/Protein Mini Kit (Qiagen, Venlo, Netherlands). One microgram of total RNA was used to synthesize cDNA using the high capacity RNA-to-cDNA kit (Life Technologies) and a C1000 Touch™ Thermal Cycler (Bio-Rad, CA, USA). For qPCR 1 μl of cDNA was used. qPCR was performed using a CFX96 Touch™ Real-Time PCR Detection System (Bio-Rad, CA, USA) and was analyzed in CFX™ Manager Software (Bio-Rad). The analysis was performed using TaqMan® probes (Thermo Fisher Scientific, Waltham, Massachusetts, USA) specific for glyceraldehyde-3-phosphate dehydrogenase (Hs03929097_g1, GAPDH), ARG1 (Hs00968979_m1), IDO (Hs00984148_m1), IL-10 (Hs00961622_m1), and TGF-β (Hs00171257_m1) genes. The expression of each gene was normalized to GAPDH. Relative gene expression was calculated by the 2–ΔΔCT method.

### Statistical Analysis

The probability of differences between HD/OC patients was assessed by Student's *t*-test (2-tailed Mann-Whitney U-test). Relationships between two parameters were investigated using Spearman's rank correlation test. The probabilities of OS were estimated using the Kaplan–Meier method (Mantel-Cox log-rank test). OS was defined as the time from the primary diagnosis till death. Patients with missing data on analyzed variables were excluded. The data are presented as medians (range). *p* < 0.05 were considered statistically significant. Statistical analyses were performed using GraphPad Prism 5.0 software (GraphPad Software, La Jolla, USA).

## Results

### Accumulation of MDSCs in EOC

Figure 1A performs the gating strategy and analysis of MDSC subsets. The frequency of M-MDSCs in the PB/PF/TT was higher in EOC patients vs. HD (0.13/0.38/0.75% vs. 0.06%, p < 0.001, *p* < 0.0001, *p* < 0.0001, respectively). In contrast, the abundance of PMN-MDSCs was elevated only in the TT vs. HD, and the level of eMDSCs was greater in the PB vs. HD (1.26% vs. 0.22% and 0.06% vs. 0.01%; *p* < 0.001, *p* < 0.01, respectively). Importantly, we revealed greater accumulation of M-MDSCs in the PF/TT vs. PB (0.38/0.75 vs. 0.13%, *p* < 0.01 and *p* < 0.001, respectively) as well as higher abundance of PMN-MDSCs in the TT vs. PB/PF (1.26 vs. 0.21/0.34%, *p* < 0.001 and *p* < 0.05, respectively). In contrast, we demonstrated greater accumulation of eMDSCs in the PB vs. PF samples (0.06 vs. 0.01%, *p* < 0.01) (Figure 1B).

### Clinical Significance of MDSCs

**Figure 2:**
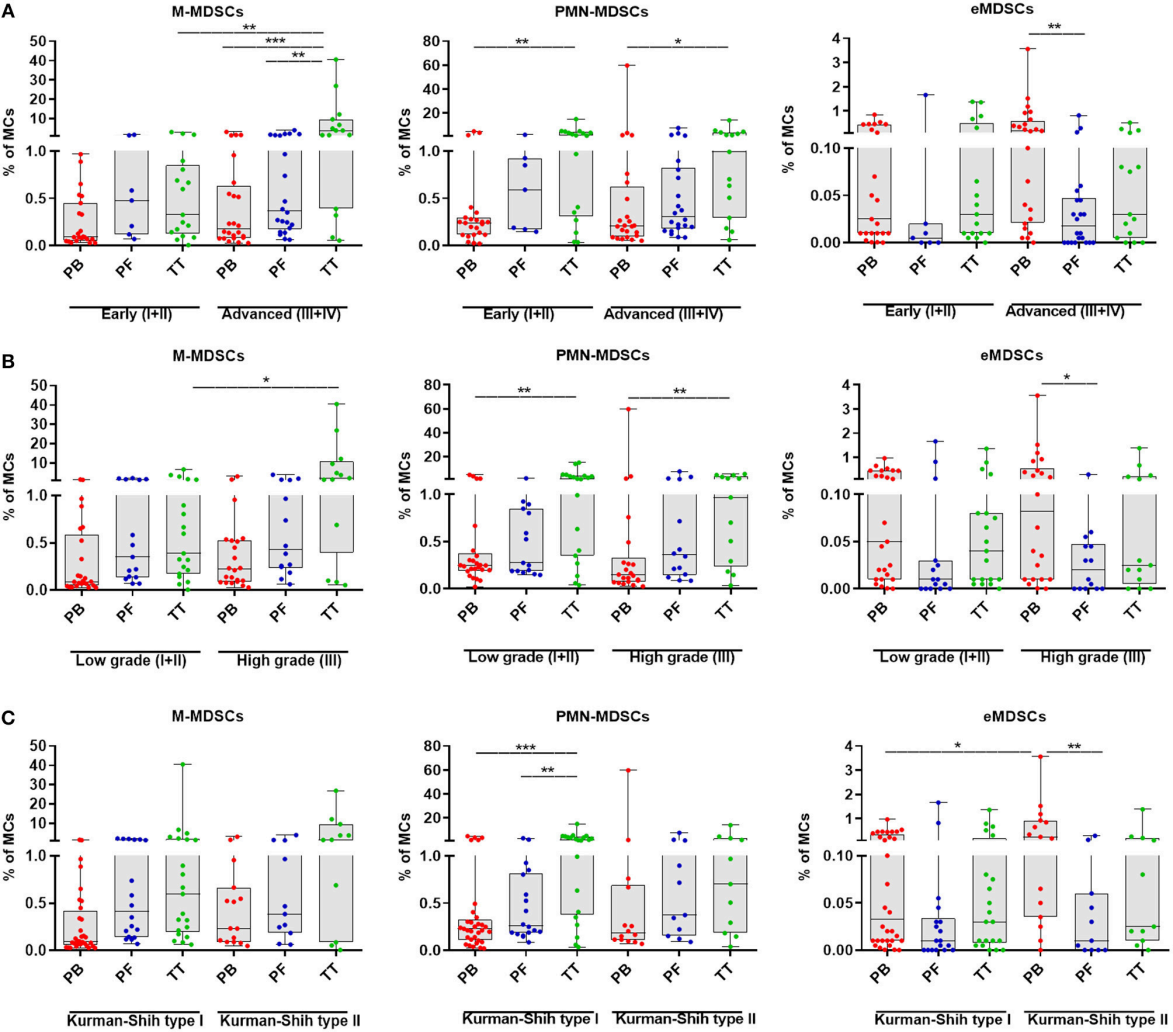
Percentage of myeloid-derived suppressor cells (MDSCs) subsets in EOC patients with different clinicopathological characteristics. Analysis of monocytic (M)-MDSCs, polymorphonuclear (PMN)-MDSCs and early-stage (eMDSCs) cells frequencies in peripheral blood (PB), peritoneal fluid (PF) and tumor tissue (TT) of EOC patients with different Federation of Gynecology and Obstetrics (FIGO) stage (early vs. advanced) **(A)**, histopathologic grading (low vs. high) **(B)** and Kurman and Shih's type (I vs. II) were examined by multicolor flow cytometry **(C)**. The frequencies of total, M-, PMN-, and eMDSCs in the PB (*n* = 47), PF (*n* = 29), and TT (*n* = 32) are presented as the percentage of MCs. The percentages of MDSC subsets from the three tumor microenvironments were determined in the same patients as described in Figure 1. Each point corresponds to an individual EOC patient. Boxes indicate the 25 to 75th percentiles. The horizontal lines within the boxes are the median values and the whiskers indicate the minimum and maximum values. Asterisks denote statistical significance (^*^p < 0.05; ^**^p < 0.01; ^***^p < 0.001).

To investigate the clinical potential of MDSC subsets, we determined its association with the patients' clinicopathological characteristics. Interestingly, the tumor progression analysis revealed higher level of tumor-infiltrating M-MDSCs in EOC patients with advanced vs. low stage and high vs. low grade (*p* < 0.01, *p* < 0.05; Figures 2A,B, respectively), whereas the abundance of PMN-/eMDSCs did not show such disparity (Figures 2A,B, respectively). However, higher accumulation of eMDSCs but not M-/PMN-MDSCs in the PB, were found in type II vs. type I (*p* < 0.05; Figure 2C). Moreover, we observed elevated levels of M-MDSCs in the TT vs. PB/PF in advanced stage (*p* < 0.001, *p* < 0.01; Figure 2A, respectively). Significantly higher percentage of PMN-MDSCs in the TT vs. PB was also revealed in all stages (*p* < 0.01 and *p* < 0.05; Figure 2A, respectively) and low/high grade (*p* < 0.01, Figure 2B). In contrast, the frequency of eMDSCs was higher in the PB vs. PF in advanced stage/high grade/type II (*p* < 0.01, *p* < 0.05, *p* < 0.01; Figures 2A–C, respectively). Besides, we observed greater accumulation of PMN-MDSCs, but not M-/eMDSCs in the TT vs. PF/PB in the type I (*p* < 0.01, *p* < 0.001, respectively; Figure 2C). When comparing patients with different histologic tumor types, we only found a significantly higher level of PMN-MDSC in the TT vs. PB in endometrioid cystadenocarcinoma (*p* < 0.05; Supplementary Figure 1A).

In accordance with the previous reports showing clinical significance of MDSCs in malignancies (18), the accumulation of notably tumor-infiltrating M-MDSCs was associated with poor clinical outcome.

### Identification of a Different Immunosuppressive Pattern Within M- and PMN-MDSCs

**Figure 3:**
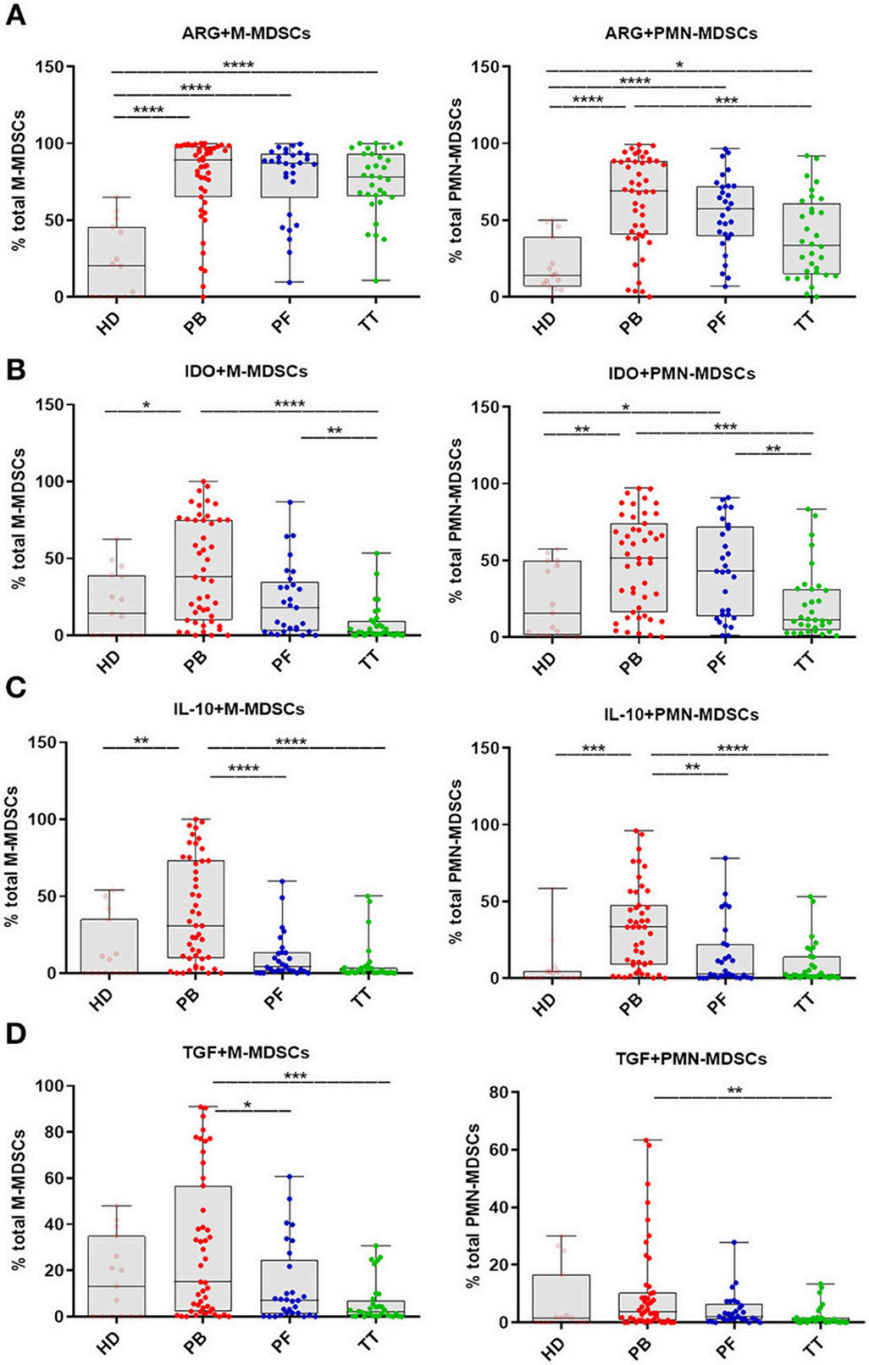
Immune pattern of monocytic (M)-MDSCs and polimorphonuclear (PMN)-MDSCs in EOC patients. The expression levels of arginase 1 (ARG1), indolamine 2,3-dioxygenase (IDO), interleukin 10 (IL-10) and transforming growth factor β (TGF-β) in MDSC subsets were assessed. Samples from the peripheral blood (PB) (*n* = 47), peritoneal fluid (PF) (*n* = 29) and tumor tissue (TT) (*n* = 32) of EOC patients and healthy donors (HD) (*n* = 15) were examined by flow cytometry. The frequencies of ARG^+^/IDO^+^/IL-10^+^/TGF-β^+^M-/PMN-MDSCs **(A–D)** are shown as the percentage of the total respective subsets **(A–D)**. The percentages of MDSC subsets from the three tumor microenvironments were determined in the same patients as described in Figure 1. Each point corresponds to an individual patient or healthy donor (HD). Boxes indicate the 25 to 75th percentiles. The horizontal lines within the boxes are the median values and the whiskers indicate the minimum and maximum values. Asterisks show statistical significance (^*^p < 0.05; ^**^p < 0.01; ^***^p < 0.001; ^****^p < 0.0001).

Guided by the reported immunosuppressive potential of MDSCs in gynecological malignancies, we analyzed intracellular expression of immune-regulatory molecules (i.e., ARG1/IDO/IL-10/TGF-β) which has been reported to play a crucial role in the function of M-/PMN-MDSCs (8). The levels of ARG+M-/PMN-MDSCs in the PB/PF/TT within the respective total subsets were higher in patients vs. HD (*p* < 0.0001 and *p* < 0.0001, *p* < 0.0001, *p* < 0.05; respectively, Figure 3A). In contrast, only frequencies of IDO+/IL-10+/M-/PMN-MDSCs in the PB but not PF/TT were significantly greater than in HD (*p* < 0.05, *p* < 0.01, and *p* < 0.01, *p* < 0.001; Figures 3B,C, respectively); we failed to observe similar changes in the abundance of TGF-β+M-/PMN-MDSCs (Figure 3D). However, we revealed higher levels of IDO+/IL-10+/TGF-β+M-/PMN-MDSCs in the PB vs. TT (*p* < 0.0001, *p* < 0.0001, *p* < 0.05 and *p* < 0.001, *p* < 0.0001, *p* < 0.01; Figures 3B–D, respectively). Additionally, accumulation of ARG+PMN-MDSCs but not ARG+M-MDSCs was greater in the PB vs. TT (*p* < 0.001; Figure 3A). Furthermore, the level of IDO+PMN-MDSCs was elevated in the PB/PF vs. TT (*p* < 0.001, *p* < 0.01; Figure 3B, respectively). Similarly, the frequencies of IDO+M-/PMN-MDSCs were higher in the PF vs. TT (*p* < 0.01; Figure 3B). Besides, higher abundance of IL-10+M-/PMN-MDSCs as well as TGF-β+M-MDSCs was demonstrated in the PB vs. PF (*p* < 0.0001, *p* < 0.01, *p* < 0.05; respectively; Figures 3C,D).

Our data indicate M-/PMN-MDSC as the subsets with immunosuppressive potential.

### Clinical Relevance of M- and PMN-MDSCs With Different Immunosuppressive Pattern

**Figure 4:**
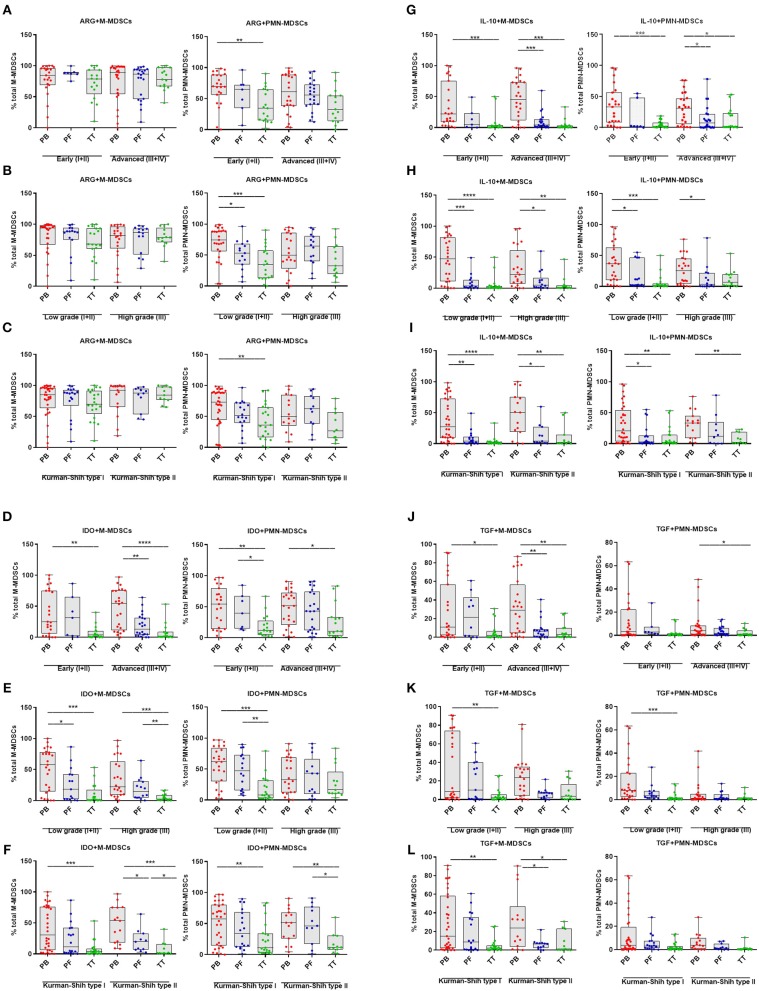
Immune pattern of monocytic myeloid derived suppressor cells (M-MDSCs) and polimorphonuclear (PMN)-MDSCs in EOC with different clinicopathological characteristics of patients. Examination of arginase 1 (ARG1) **(A–C)**, indolamine 2,3-dioxygenase (IDO) **(D–F)**, interleukin 10 (IL-10) **(G–I)**, and transforming growth factor β (TGF-β) **(J–L)**-expressing M-/PMN-MDSCs in EOC patients with different clinicopathological characteristics. Samples from the peripheral blood (PB) (*n* = 47), peritoneal fluid (PF) (*n* = 29), and tumor tissue (TT) (*n* = 32) of patients with different Federation of Gynecology and Obstetrics (FIGO) stage (early vs. advanced) **(A,D,G,J)**, histopathologic grading (low vs. high) **(B,E,H,K)** and Kurman and Shih type (I vs. II) **(C,F,I,L)** were examined by flow cytometry. The frequencies of ARG^+^/IDO^+^/IL-10^+^/TGF-β^+^M-/PMN-MDSC subsets **(A–L)** are shown as the percentage of the total respective subsets. The percentages of MDSC subsets from the PB/PF/TT were assessed in the same patients as described in Figure 1. Each point corresponds to an individual patient. Boxes indicate the 25 to 75th percentiles. The horizontal lines within the boxes are the median values and the whiskers indicate the minimum and maximum values. Asterisks show statistical significance (^*^p < 0.05; ^**^p < 0.01; ^***^p < 0.001; ^****^p < 0.0001).

Next, we investigated the clinical potential of MDSCs with a different immunosuppressive profile. We showed higher frequencies of ARG+PMN-MDSCs in the PB vs. TT in early stage/low grade/type I of EOC (*p* < 0.01, *p* < 0.0001, *p* < 0.01; respectively; Figures 3A–C) and higher level of above subset in the PB vs. PF in low grade (*p* < 0.05, Figure 3B). In contrast, patients showed no differences in the frequency of ARG+M-MDSCs in the PB/PF/TT in all stages, grades and types of EOC (Figures 4A–C). However, we observed elevated level of IDO+M-MDSCs in the PB vs. TT in the early/advanced stages (*p* < 0.01, *p* < 0.0001; respectively, Figure 4D), low/high grades (*p* < 0.001, Figure 4E) and types I/II (*p* < 0.001, *p* < 0.05; Figure 4F).

Similarly, we demonstrated higher accumulation of IDO+PMN-MDSCs in the PB vs. TT in early/advanced stages (*p* < 0.01, *p* < 0.05; respectively; Figure 4D), low grade (*p* < 0.001, Figure 4E) and types I/II (*p* < 0.01, Figure 4F). Besides, the abundance of IDO+M-MDSCs was higher in the PB vs. PF in advanced stage/low grade/type I (*p* < 0.01, *p* < 0.05, *p* < 0.05; respectively; Figures 4D–F) and higher in the PF vs. TT in high grade/type II (*p* < 0.01, *p* < 0.05; respectively; Figures 4E,F). Similarly, greater accumulation of IDO+PMN-MDSCs in the PF vs. TT was observed in early stage/low grade/type II (*p* < 0.05, *p* < 0.01, *p* < 0.05; respectively, Figures 4D–F).

The examined expression of IL-10+M-MDSCs revealed its significant elevation in the PB vs. TT in all stages (*p* < 0.001, Figure 4G), grades (*p* < 0.0001, *p* < 0.01, respectively; Figure 4H) and types of cancer (*p* < 0.0001, *p* < 0.01, respectively; Figure 4I) as well as higher in the PB vs. PF in advanced stage (*p* < 0.001; Figure 4G), low/high grades (*p* < 0.001, *p* < 0.05, respectively; Figure 4H) and types I/II (*p* < 0.01, *p* < 0.05, respectively; Figure 4I). Similarly, we observed higher abundance of IL-10+PMN-MDSCs in the PB vs. TT in early/advanced stages (*p* < 0.001, *p* < 0.05, respectively; Figure 4G), low grade (*p* < 0.001, Figure 4H) and types I/II (*p* < 0.01, Figure 4I) as well as greater accumulation in the PB vs. PF in advanced stage/low grade/type I (*p* < 0.05, Figures 4G–I).

An analysis of TGF-β+M-MDSCs revealed its significantly higher level in the PB vs. TT in early/advanced stage (*p* < 0.05, *p* < 0.01; Figure 4J, respectively), low grade (*p* < 0.01; Figure 4K) and type I/II (*p* < 0.01, *p* < 0.05; Figure 4L, respectively) as well as higher accumulation of this population in the PB vs. PF in advanced stage/type II (*p* < 0.01, *p* < 0.05, respectively; Figures 4J–L). Similarly, enhanced level of TGF-β+PMN-MDSCs in the PB vs. TT was demonstrated in advanced stage/low grade (*p* < 0.05 and *p* < 0.001, respectively; Figures 4J,K).

When comparing patients with various histologic types, we observed a significantly greater frequency of IDO+M-/PMN-MDSCs in the PB vs. TT in serous type (*p* < 0.001, *p* < 0.05 Supplementary Figure 1C, respectively). Additionally, the level of IL-10+M-MDSCs was higher in the PB vs. PF/TT in endometrioid type (*p* < 0.05, *p* < 0.01; Supplementary Figure 1D, respectively). Besides, higher accumulation of above subset was observed in the PB vs. TT in serous type (*p* < 0.01; Supplementary Figure 1D). We revealed neither differences between levels of other MDSC subsets tested in the patients with various histologic types of EOC (Supplementary Figures 1B,D,E) nor disparities in the distribution of ARG1+/IDO+/IL-10+/TGF-β+M-/PMN-MDSCs in the patients with a different clinicopathological characteristic (Figures 4A–L).

The strong accumulation of potential immunosupressive ARG+/IDO+/IL10+/TGF-β+M-/ PMN-MDSCs may be tumor-progression independent and may pose a significant impediment to the efficacy of anti-tumor responses elicited by (immuno)therapy.

### Profile of Immunosuppressive Mediators and Its Correlation With the MDSCs

**Figure 5:**
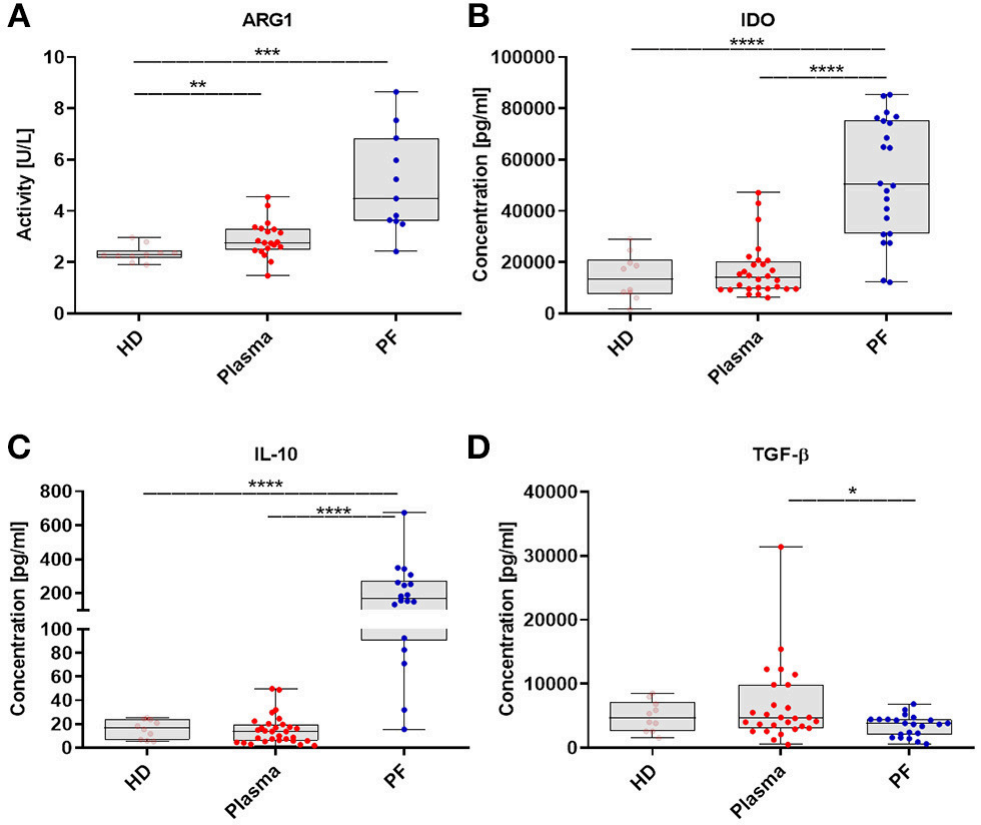
The myeloid derived suppressor cells (MDSCs)-related immunosuppressive mediators pattern in EOC patients. Immunosuppressive factors in the plasma and peritoneal fluid (PF) from EOC patients and healthy donors (HD) were assayed. Arginase 1 (ARG1) activity was assessed in healthy donors (HD) (*n* = 10), plasma (*n* = 20) and peritoneal fluid (PF) (*n* = 11) using QuantiChrom™ Arginase Assay Kit **(A)**. The concentrations of indolamine 2,3-dioxygenase (IDO) (HD, *n* = 10; plasma, *n* = 28; PF, *n* = 22) **(B)**, interleukin 10 (IL-10) (HD, *n* = 10; plasma, *n* = 30; PF, *n* = 18) **(C)**, transforming growth factor β (TGF-β) (HD, *n* = 10; plasma, *n* = 27; PF, *n* = 22) **(D)**, were determined using enzyme-linked immunosorbent assay (ELISA). The levels of factors were analyzed in the same available samples from patients which are described in Figure 1. Each point corresponds to an individual patient or a healthy donor (HD). Boxes indicate the 25 to 75th percentiles. The horizontal lines within the boxes are the median values and the whiskers indicate the minimum and maximum values. Asterisks present statistical significance (^*^p < 0.05; ^**^p < 0.01; ^***^p < 0.001; ^****^p < 0.0001).

In order to more deeply characterize immune-regulatory activity of MDSCs, we expanded our analysis of MDSCs to include measurement of crucial immunosupressive mediator levels. The levels of factors were analyzed in the same available samples from patients described in Figure 1. The activity of ARG1 was higher in the patients plasma/PF vs. HD (*p* < 0.01, *p* < 0.001, respectively; Figure 5A). Besides, we observed enhance levels of IDO and IL-10 in the PF vs. plasma/HD (*p* < 0.0001; Figures 5B,C). In contrast, concentration of TGF-β was greater in the plasma vs. PF (*p* < 0.05; Figure 5D).

Further correlation analysis confirmed a positive correlation of the level of TGF-β in the plasma with frequency of ARG+M-MDSCs in the PF (*p* = 0.02; Supplementary Figure 2B). Additionally, activity level of ARG1 in the PF was positively correlated with the abundance of PMN-MDSCs in the PF (*p* = 0.02; Supplementary Figure 2C); as well as high level of ARG1 in the plasma was associated with greater frequency of TGF-β+/IDO+/IL-10+PMN-MDSCs in the PF (*p* = 0.02, *p* = 0.006, *p* = 0.04; Supplementary Figures 2D–F). None of the other factors tested was significantly associated with the frequency of MDSC subsets (data not shown).

Finally, gene expression analysis of ARG1/IDO/IL-10/TGF-β in MCs isolated from the PB/PF/TT revealed significant differences in expression of these genes in the PF but not in the PB/TT. Significantly higher ARG1/IL-10/TGF-β than IDO gene expression was observed in the PF (*p* < 0.001, *p* < 0.01, *p* < 0.01, respectively). We did not detect the mRNA expression levels of IL-10 in MCs from the PB as well as ARG1 in MCs from the TT (Supplementary Figure 2A). We failed to observe any significant correlations between levels of mRNA and MDSC subsets (data not shown).

Summarizing, we demonstrated that the immune-regulatory mediators milieu in which EOC disease progress is skewed toward immunosupressive phenotype and will likely adversely impact the anti-tumor immune responses by accumulation of different M-/PMN-MDSCs.

### Declines of Blood-Circulating and Tumor-Infiltrating M-MDSCs Are Associated With Favorable Survival

**Figure 6:**
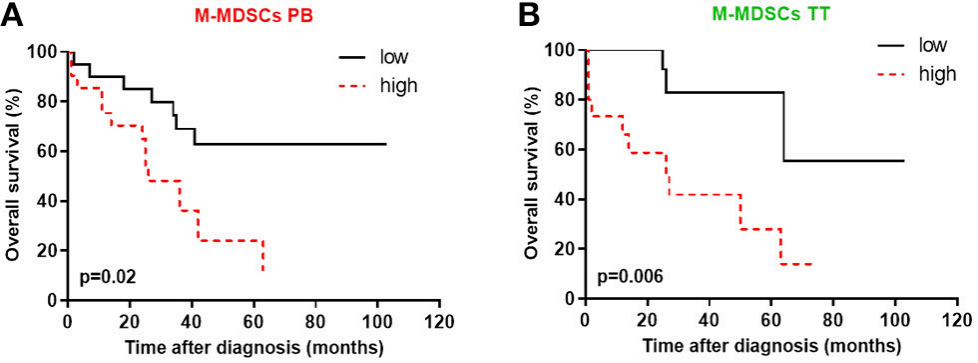
High levels of blood circulating and tumor-infiltrating monocytic myeloid derived suppressor cells (M-MDSCs) are associated with poor survival. The percentages of MDSC subsets in the peripheral blood (PB) and tumor tissue (TT) were determined in the same patients as described in Figure 1. The overall survival (OS) values of the patients with high/low M-MDSCs in the PB **(A)** and high/low M-MDSCs in the TT **(B)** among mononuclear cells (MCs) are shown as Kaplan-Meier curves. Univariate analysis of OS in patients with EOC was performed using Kaplan Meier method based on MDSCs percentage; solid black line: values below median, intermittent red line: values above median. Statistical analysis of survival was performed by the log-rank (Mantel-Cox) analysis. Results were considered significant at *p* < 0.05.

Because, prior reports have suggested that MDSCs accumulation is negatively correlated with cancer patient survival (18), we inquired whether MDSC subsets in the PB/PF/TT predict survival in EOC patients. As shown in Figures 6A,**B**, patients with a high frequency of M-MDSCs in the PB and TT significantly decreased survival (*p* = 0.02 and *p* = 0.006, respectively). Of note, no correlation between OS and the distribution of M-MDSCs in the PF and both PMN-/eMDSC subsets existed (Supplementary Figures 3A–C).

## Discussion

The tumor milieu is a main battle arena between tumor cells and the immune cells which can be corrupted by non-transformed cells (4, 27). The interaction between tumor cells and host immune cells causes immunoediting, promotes immune escape, and finally results in tumor metastasis and relapse (21, 28, 29). Thus, cancer is considered as a complex disease in which the interaction between the immune cells and tumor cells determines the outcome of the disease and thus the immune system components constitute potential therapeutic targets. Owing to our and other studies it is now well-established that there are several tumor mediated immunosuppressive networks operating in EOC (18, 21–24, 30, 31). One of these networks involves accumulation of MDSCs, which has an important role in poor clinical outcome and concomitantly suppression of immune responses in cancer provide rationale to attempt to curtail/eradicating these cells.

However, while MDSCs have been extensively studied in mouse cancer models, the human studies on MDSCs was in its infancy for many years owing to the paucity on consensus on immunophenotyping (32). Additionally, in most studies, MDSCs were only analyzed in PB neither TT nor other human body fluids (8). Notably, the favorable vs. detrimental role of the TIME immune contexture might depend on the location of immune cells, which can be related with miscellaneous clinical outcomes according to where these cells are assessed (33). Therefore, further clinical study design on MDSCs should embrace multivariate approach.

In 2016, recommendations for the characterization of human MDSCs were proposed (17). To the best of our knowledge, neither comparative analysis of the three MDSC subsets in the three TIME nor their immunosupressive pattern and clinical importance in human cancer is available to date. Our previous research explored for the first time the clinical impact of tumor-infiltrating MDSCs and revealed that patients whose OC samples were CD33high (by immunohistochemistry staining) had decreased OS compared to patients with CD33low samples (21). These observations provide rationale to extend the previous findings. To address this issue we examined frequency, immunosupressive pattern and MDSC-related immunosupressive factors of the three MDSC subsets in the three TIME of EOC and integrated this analysis with patients' clinicopathological characteristic.

We found that M-MDSCs in the three tumor milieu are elevated in the patients vs. HD. These findings support those of several other groups (32, 34). Whilst, only TT but not PF/PB PMN-MDSCs frequency significantly increased compared to HD. This is in agreement with previous findings which showed similar level of circulating HLA-DR^−/low^CD11b^+^CD14^−^CD15^+^PMN-MDSCs between lung cancer patients and HD (34). In contrast, Najjar et al. revealed significantly increased number of PMN-MDSCs in the PB/TT of renal cell carcinoma (RCC) vs. control (35). The reason for the partial discrepancies reported for blood-circulating PMN-MDSCs in EOC may be related with the patient populations studied, source/processing of PBMCs, antibody cocktails used and the high heterogeneity of this subset. Nevertheless, the analysis of tumor-infiltrating M-/PMN-MDSCs revealed strong abundance of these subsets vs. circulating counterparts. Thus, data confirms that the tumor site is characterized by the formidable accumulation of potentially immunosuppressive MDSCs (7). Contrary to M-/PMN-MDSCs, little is known about source/function/identity of eMDSCs. We found a significantly greater level of eMDSCs in the blood vs. PF/HD. In contrast, previous study showed similar accumulation of circulating eMDSCs in HD and head and neck cancer which possessed indiscernible immunosuppressive potential and no clinical relevance (32). However, our results are partially in accordance with observations of Najjar's group, which demonstrated elevated level of eMDSCs in the PB/TT of RCC. It is worth notifying that partial disparity in our observations and another results about eMDSCs may be related with the different mAb used for immunophenotyping.

Integrating above findings with clinical data, we demonstrated that high abundance of tumor-infiltrating M-MDSCs but not PMN-/eMDSCs was associated with increasing tumor stage and grade. While several studies revealed a correlation between M-MDSC in the tumor lesions and patient outcomes, to our knowledge, ours is the first to show a positive correlation between accumulation of tumor-infiltrating M-MDSCs and both grade/stage of EOC. Interestingly, Wu et al. demonstrated increased frequency of circulating CD14^+^HLA-DR^−/low^ cells in EOC and its correlation with stage, but not grade or histological type (23). However, besides observed tendency to higher stage/grade/type-dependent abundance of circulating M-MDSCs, our findings imply no significant disparity. It is worth notifying, that Toor's group demonstrated that circulating PMN-MDSCs frequency correlates with tumor grade in colorectal cancer (36) and Gielen's group showed grade-dependent accumulation of PMN-MDSCs in glioblastoma (37). However, we failed to show similar significant changes in the accumulation of circulating PMN-MDSCs in EOC. Interestingly, we observed that blood-circulating eMDSCs but not M-/PMN-MDSCs correlated with the more aggressive type II of EOC, although recent study of Lang et al. demonstrated the level of circulating eMDSCs was not associated with patient outcome (32). However, another studies showed that higher level of circulating HLA-DR^−^CD11b^+^CD33^+^ population was correlated with advanced stages and lymph node metastases (38) and greater abundance of circulating Lin^−/low^HLA-DR^−^CD11b^+^CD33^+^ cells was correlated with stage and tumor metastasis but not primary tumor size in colorectal cancer (39). Thus, above observations confirm tremendous heterogeneity of the clinical relevance of MDSCs in human cancers.

Given the previous extensive reports about immunosuppressive activity of M-/PMN-MDSCs (6, 14, 28), we further examined immunosuppressive pattern of these two main abundant subsets in EOC. Our study reveals a previously undescribed picture of extreme immune dysfunction in disease. Those analyses showed a significant increase in the frequency of ARG-expressing M-/PMN-MDSCs in the patients in all three TME vs. HD. ARG is a key molecule that mediates the immunosupressive action of MDSCs via deplete L-arginine from TIME and inhibit T-cell functions (40, 41). Importantly, we also detected a higher frequency of circulating IDO^+^/IL-10^+^M-/PMN-MDSCs in the patients vs. HD. While, IDO restrains the activation of effector T cells via depletion of tryptophan and favors activation/differentiation of Foxp3^+^ regulatory T cells through production of kynurenine (42), it is postulated that IL-10 synergistically promoted the expansion of MDSCs by upregulation of STAT3 in combination with IL-6 (23). Although we observed similar accumulation of TGF-β1^+^M-/PMN-MDSCs in the PB/PF/TT vs. HD, we revealed higher abundance of these subsets in the blood vs. tissue lesions. Observed tendency to higher accumulation of ARG/IDO/IL-10/TGF-β-expressing M-/PMN-MDSCs in the blood of patients vs. PF/TT, indicating formidable upregulated immunosupressive potential of the blood-circulating subsets. Circulating inflamed/immunosuppressive M-/PMN-MDSCs can be considered as to be immunologically “hot” cells in ovarian cancer (43). There is only one article reporting the immunosuppressive activity of MDSCs in EOC; these findings showed, that accumulation and suppressive activity of M-MDSCs depend on the PF-derived IL-6/IL-10 (23). Recent work also demonstrated mature PMN-MDSCs as the most suppressive subset in head and neck and urogical cancer. The same experiments revealed immature CD11b^−^CD16^−^ PMN-MDSCs as less suppressive than mature counterparts (32). Additionally, few studies also demonstrated PMN-MDSCs as less immunosuppressive subset than M-MDSCs (40). These discrepancies are not surprising and can be explained by using different markers for identifying MDSC subsets and/or formidable inter-/intratumor heterogeneity (44, 45). Furthermore, the appearance of this phenomenon may be related to PMN-MDSC subset stability. The M-MDSCs were found to be more resistant to the thawing compared to the PMN-MDSCs (46). Besides, recent report demonstrated that only fresh CD15-HLA-DR-Lin- cells were ARG1+, but lost its expression after thawing (47). Taking into consideration all above, the results should be interpreted with caution. Our findings offer rationale for further research addressing over this affair in human cancer. Of note, our data indicate M-/PMN-MDSCs with the immunosuppressive potential and immunomonitoring/eradication of these subsets may be the holy grail of EOC immunology.

Given the widespread immunosuppressive network that we found in MDSCs from EOC, we explored its clinical significance. Data revealed no significant dependency in the accumulation of ARG/IDO/IL-10/TGF-β-expressing M-/PMN-MDSCs in the three EOC milieu and the disparity of patients' clinicopathological characteristic. It seems probable that abundance of above factors may be tumor-progression independent. However, our data showed differences in the abundance of immunosuppressive MDSCs depending on the TIME in different clinical manifestation of disease. Recent data reported positive correlation of circulating PMN-MDSCs in the RCC with tumor grade; however the same study finds no corellation between perenchymal MDSCs level and grade (35). We speculate that development of immunosupresive properties in MDSCs could occur when these cells are placed in a proper cytokine/chemokine/growth factor milieu. In the future, it is tantalizing to speculate that EOC treatment might be tailored to cellular make-up, based on immunosuppressive repertoire.

In the light of our previous findings about intercellular communication in EOC driven by complex/dynamic network of immunosupresive mediators we examined pivotal MDSC-related factors (9, 11–13, 21). Indeed, we demonstrated that level of TGF-β in the plasma positively correlate with the frequency of ARG^+^M-MDSCs in the PF. Moreover, the higher activity of ARG1 in the PF correlate with greater abundance of PMN-MDSC subset in the PF. Additionally, the activity of ARG1 in the plasma positively correlate with the frequencies of TGF-β^+^/IDO^+^/IL-10^+^PMN-MDSCs in the PF. To our knowledge, only one study measured arginase activity in the plasma of EOC patients but none assessed its activity in the PF. Nishio et al. observed higher arginase activity in EOC patient plasma vs. HD as well as its correlation with the plasma IL-8 levels (48). Our observations indicating that EOC-derived ARG1 may be involved in the enhancement of immunosuppressive activity via an increase of abundance of immunosupressive cell subsets. Moreover, our data suggest that different mediators, depending on the location in TIME promote accumulation of different MDSC subsets. Although we did not find correlation between mRNA expression of *ARG1/IDO/IL-10/TGF-*β in the PB/PF/TT, which may be due to small representative samples, we observed upregulation of *ARG1, IL-10*, and *TGF-*β expression level compared with the level of *IDO* in the PF. All of above events lead to “perfect storm” ideally suited to tumor immune escape to create metastasis-promoting TIME. Additionally, current data indicate that PF-derived mediators may play an important role in immune modulation of EOC milieu and seem to be one of the most likely players for MDSC-mediated support of tumor growth, which is not surprising, given that our previous work indicates that numerous factors are involved in the formation of ascites and promote dissemination of EOC cells (9, 49, 50). Indeed, it may be reasonable to consider the use of inhibitors against PF-derived immunosupressive mediators to restrain cancer metastasis. Data suggest that targeting the mediators or/and immune cells can be beneficial across different malignancies and could complement other therapeutic options.

Interestingly, we revealed that high accumulation of blood-circulating and tumor-infiltrating M-MDSCs is negatively correlated with patient survival. Meanwhile, we failed to observe any correlations of PMN-/eMDSCs with the OS of patients. Most publications have indicated correlations between improved cancer patients survival and downregulation of blood-circulating M-MDSCs (38, 51–54), which is consistent with our data. It is worth notifying, that some authors demonstrated a predictive role of blood-circulating PMN-MDSCs but not M-/eMDSCs, which is partially consistent with our results (32). Indeed, current study revealed the prognostic value of M-MDSCs for EOC patients. It is worth notifying, that our work ascribes a pivotal role to the “liquid biopsy” concept to determine disease prognosis and immunomonitoring which was recently described by our group (18).

Our study is not without limitations and thus need to be interpreted with caution. Firstly, the use of cryopreserved PBMCs might not reflect the actual immune milieu *in vivo*, for example due to differences in susceptibility to cryopreservation between MDSC subsets. However, this method has the logistical advantage of facilitating transport/batch analysis and thus is crucial for immune-monitoring of multicentre studies. Secondly, the enrolled patients were limited in number which may have led to type II error in some instances, e.g., inability to discern correlations between mRNA levels of immunosupresive factors and MDSCs. Thirdly, our clinical studies were conducted in a single institution, thus we cannot draw definitive conclusions regarding the clinical significance/prognostic value of MDSCs in EOC. Thus, to validate our clinical findings, a multi-institutional investigation is warranted.

In brief, our results indicate M-MDSCs as the subset with potential the highest clinical relevance. Looking forward, targeting various aspects of TIME, including MDSCs might be a landmark where its suppressive and tumor-fostering immune system is reprogrammed/switched off, its faulty blood content is normalized and cancer cells are destroyed.

## Data Availability

All datasets generated for this study are included in the manuscript and/or the Supplementary Files.

## Author Contributions

KO, IW, and JK: conception and design; KO, AC, and, AW: development of methodology; MoB, MaB, RT, WB, IW, and JK: acquisition of data (acquired and managed patients, provided facilities, etc.); KO, AC, AW, IW, and JK: analysis and interpretation of data (e.g., statistical analysis, biostatistics, computational analysis); KO, IW, and JK: writing, review, and/or revision of the manuscript: MaB, MoB, RT, and WB: administrative, technical, or material support (i.e., reporting or organizing data, constructing databases); JK and IW: study supervision.

### Conflict of Interest Statement

The authors declare that the research was conducted in the absence of any commercial or financial relationships that could be construed as a potential conflict of interest.

## Supplementary Material

Supplementary Figure 1 Frequency of MDSC subsets in EOC patients with different histologic type. Analysis of monocytic (M)-MDSCs, polymorphonuclear (PMN)-MDSCs and early-stage eMDSCs cells frequencies in peripheral blood (PB) (*n* = 47), peritoneal fluid (PF) (*n* = 29) and tumor tissue (TT) (*n* = 32) of epithelial ovarian cancer (EOC) patients with different histological type including serous (S), endometrioid (E), mucinous (M) cystadenocarcinoma and undifferentiated carcinoma (U) **(A)**. The percentage of ARG1^+^M-/PMN-MDSCs **(B)** IDO^+^M-/PMN-MDSCs **(C)**, IL-10^+^M-/PMN-MDSCs **(D)**, TGF-β^+^M-/PMN-MDSCs **(E)** in the PB/PF/TT of EOC patients with M/E/S/U histological type. The frequency of MDSC subsets was examined by multicolor flow cytometry and they are presented as the percentage of the MCs **(A)** or the percentage of the total respective subsets **(B–E)**. Each point corresponds to an individual patient. Boxes indicate the 25 to 75th percentiles. The horizontal lines within the boxes are the median values and the whiskers indicate the minimum and maximum values. Asterisks represent statistical significance (^*^*p* < 0.05; ^**^*p* < 0.01; ^***^*p* < 0.001).

Supplementary Figure 2 The myeloid derived suppressor cells (MDSCs)-related immunosuppressive mediators pattern and its correlation with the pattern of MDSC subsets in EOC patients. RNA from mononuclear cells (MCs) from peripheral blood (PB), PF and tumor tissue (TT) of eight patients with EOC were isolated and the mRNA expression gene levels of ARG1, IDO, IL-10, and TGF-β was determined using quantitative polymerase chain reaction (qPCR). Data were normalized to the GAPDH (fold change) **(A)**. The correlation of TGF-β in the plasma vs. ARG+M-MDSCs in the PF **(B)**, level of ARG1 activity vs. level of polymorphonuclear (PMN)-MDSCs in the PF **(C)**, level of ARG1 in the plasma vs. TGF-β+PMN-MDSCs **(D)**, IDO+PMN-MDSCs **(E)** and IL-10+PMN-MDSCs in the PF **(F)** was performed. The levels of factors were analyzed in the same available samples from patients which are described in Figure 1. Each point corresponds to an individual patient or a healthy donor (HD). Boxes indicate the 25 to 75th percentiles. The horizontal lines within the boxes are the median values and the whiskers indicate the minimum and maximum values. Asterisks present statistical significance (^**^*p* < 0.01; ^***^*p* < 0.001); n.d, not detected.

Supplementary Figure 3 Kaplan-Meier overall survival curves for patients with EOC. The percentages of MDSC subsets in the three tumor microenvironments including peripheral blood (PB), peritoneal fluid (PF) and tumor tissue (TT) were determined in the same patients as described in Figure 1. The overall survival (OS) values of the patients with high/low M-MDSCs **(A)** high/low PMN-MDSCs **(B)** and high/low eMDSCs **(C)** among mononuclear cells (MCs) are shown as Kaplan-Meier curves. Univariate analysis of OS in patients with EOC was performed using Kaplan Meier method based on MDSCs percentage; solid black line: values below median, intermittent red line: values above median. Statistical analysis of survival was performed by the log-rank (Mantel-Cox) analysis. Results were considered significant at *p* < 0.05.

Supplementary Table 1 Characteristic of the patients and healthy donors.
