# Supplementary figures and images for: Clinical Relevance and Immunosuppressive Pattern of Circulating and Infiltrating Subsets of Myeloid-Derived Suppressor Cells (MDSCs) in Epithelial Ovarian Cancer
Source: Front Immunol. 2019 Apr 3;10:691. doi: 10.3389/fimmu.2019.00691 (PMC6456713; doi:10.3389/fimmu.2019.00691)

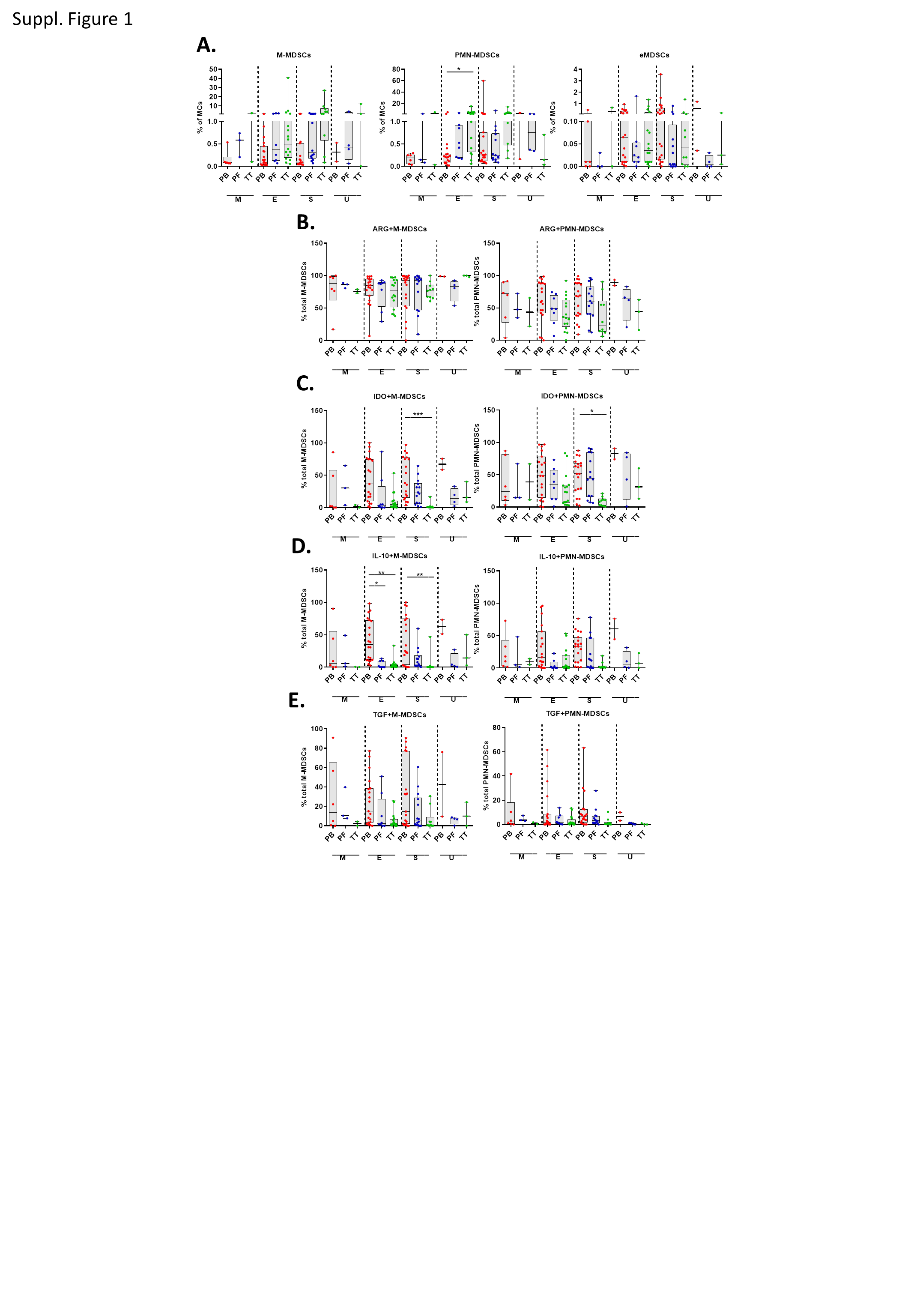

Supplement: Supplementary Figure 1 — Frequency of MDSC subsets in EOC patients with different histologic type. Analysis of monocytic (M)-MDSCs, polymorphonuclear (PMN)-MDSCs and early-stage eMDSCs cells frequencies in peripheral blood (PB) (n = 47), peritoneal fluid (PF) (n = 29) and tumor tissue (TT) (n = 32) of epithelial ovarian cancer (EOC) patients with different histological type including serous (S), endometrioid (E), mucinous (M) cystadenocarcinoma and undifferentiated carcinoma (U) (A). The percentage of ARG1+M-/PMN-MDSCs (B) IDO+M-/PMN-MDSCs (C), IL-10+M-/PMN-MDSCs (D), TGF-β+M-/PMN-MDSCs (E) in the PB/PF/TT of EOC patients with M/E/S/U histological type. The frequency of MDSC subsets was examined by multicolor flow cytometry and they are presented as the percentage of the MCs (A) or the percentage of the total respective subsets (B–E). Each point corresponds to an individual patient. Boxes indicate the 25 to 75th percentiles. The horizontal lines within the boxes are the median values and the whiskers indicate the minimum and maximum values. Asterisks represent statistical significance (*p < 0.05; **p < 0.01; ***p < 0.001). [file Image_1.TIF]

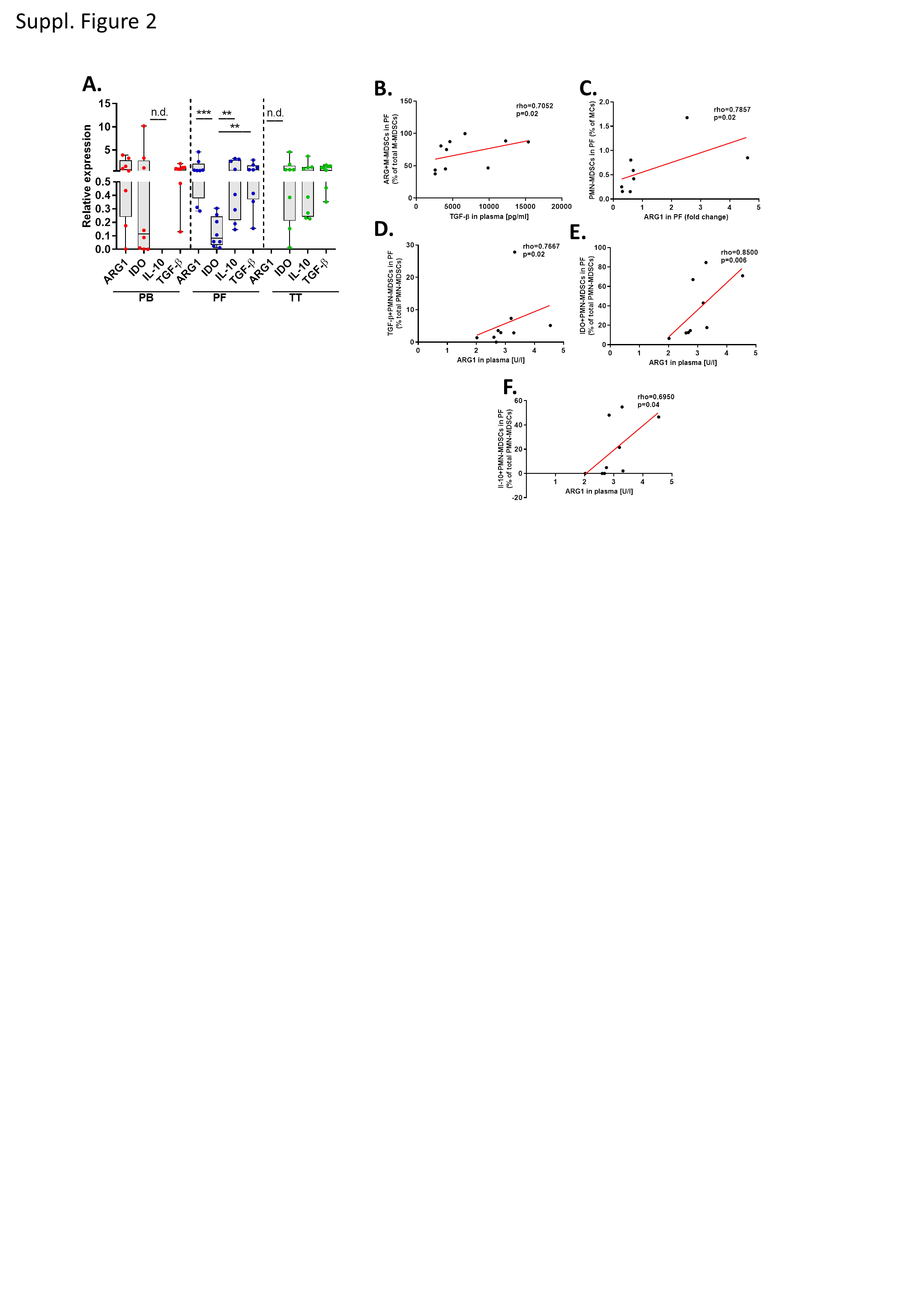

Supplement: Supplementary Figure 2 — The myeloid derived suppressor cells (MDSCs)-related immunosuppressive mediators pattern and its correlation with the pattern of MDSC subsets in EOC patients. RNA from mononuclear cells (MCs) from peripheral blood (PB), PF and tumor tissue (TT) of eight patients with EOC were isolated and the mRNA expression gene levels of ARG1, IDO, IL-10, and TGF-β was determined using quantitative polymerase chain reaction (qPCR). Data were normalized to the GAPDH (fold change) (A). The correlation of TGF-β in the plasma vs. ARG+M-MDSCs in the PF (B), level of ARG1 activity vs. level of polymorphonuclear (PMN)-MDSCs in the PF (C), level of ARG1 in the plasma vs. TGF-β+PMN-MDSCs (D), IDO+PMN-MDSCs (E) and IL-10+PMN-MDSCs in the PF (F) was performed. The levels of factors were analyzed in the same available samples from patients which are described in Figure 1. Each point corresponds to an individual patient or a healthy donor (HD). Boxes indicate the 25 to 75th percentiles. The horizontal lines within the boxes are the median values and the whiskers indicate the minimum and maximum values. Asterisks present statistical significance (**p < 0.01; ***p < 0.001); n.d, not detected. [file Image_2.TIF]

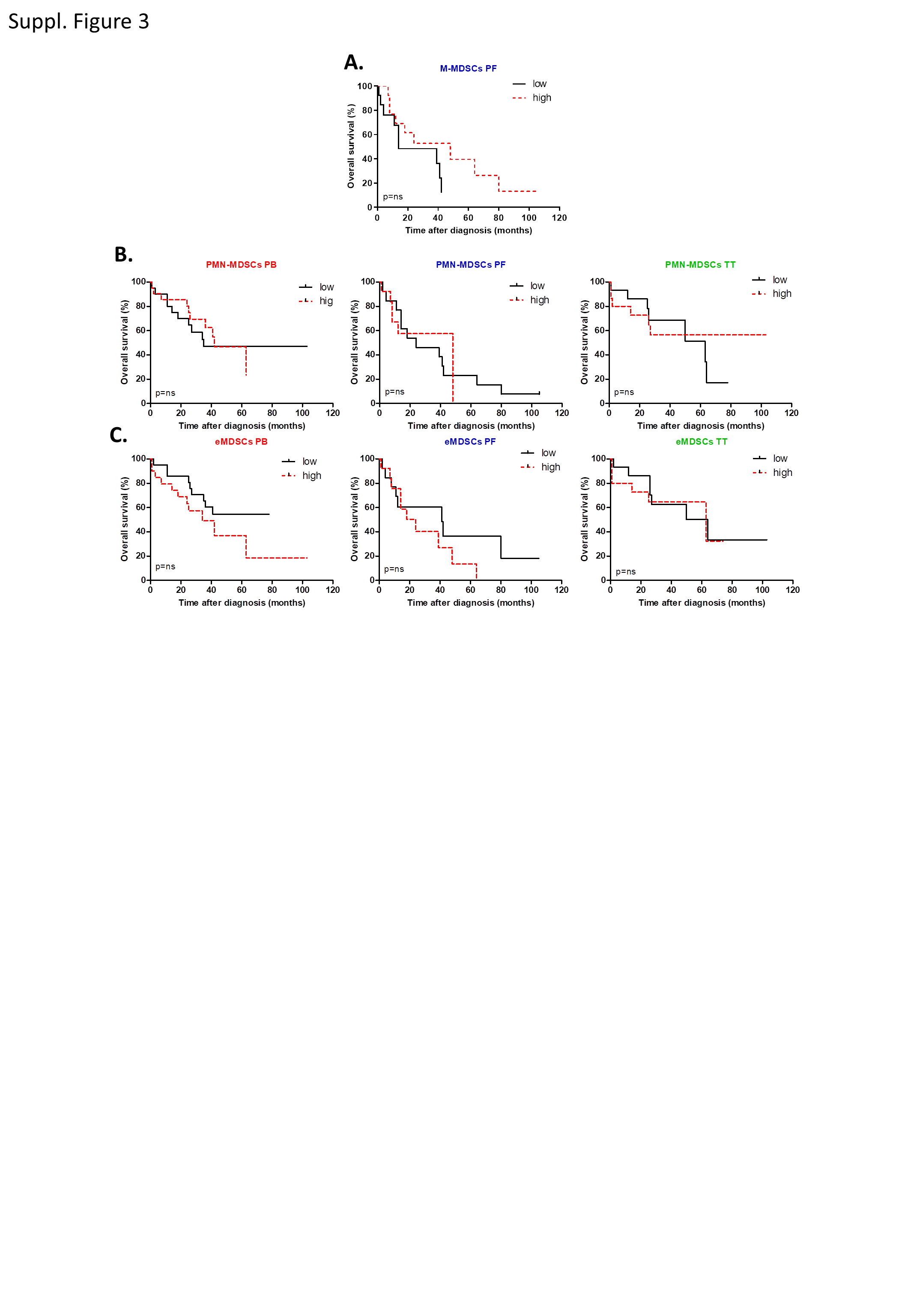

Supplement: Supplementary Figure 3 — Kaplan-Meier overall survival curves for patients with EOC. The percentages of MDSC subsets in the three tumor microenvironments including peripheral blood (PB), peritoneal fluid (PF) and tumor tissue (TT) were determined in the same patients as described in Figure 1. The overall survival (OS) values of the patients with high/low M-MDSCs (A) high/low PMN-MDSCs (B) and high/low eMDSCs (C) among mononuclear cells (MCs) are shown as Kaplan-Meier curves. Univariate analysis of OS in patients with EOC was performed using Kaplan Meier method based on MDSCs percentage; solid black line: values below median, intermittent red line: values above median. Statistical analysis of survival was performed by the log-rank (Mantel-Cox) analysis. Results were considered significant at p < 0.05. [file Image_3.TIF]
