# Supplementary material for: Clinical Relevance and Immunosuppressive Pattern of Circulating and Infiltrating Subsets of Myeloid-Derived Suppressor Cells (MDSCs) in Epithelial Ovarian Cancer
Source: Front Immunol. 2019 Apr 3;10:691. doi: 10.3389/fimmu.2019.00691 (PMC6456713; doi:10.3389/fimmu.2019.00691)
Supplement: Supplementary Table 1 — Characteristic of the patients and healthy donors. [file Table_1.DOCX]

**Supplementary Table 1.Characteristic of the patients and healthy donors**

| **Variables** | **EOC** | | | **HD** |
| --- | --- | --- | --- | --- |
| **Material (type)** | **PB** | **PF** | **TT** | **PB** |
| **Subjects (n)** | **47** | **29** | **32** | **15** |
| **Age median, years (range)** | **60 (20-86)** | **60 (20-86)** | **56 (20-85)** | **57 (34-64)** |
| **FIGO stage, n (%)** | | | |  |
| **Early** | **23 (48.9%)** | **7 (24.1%)** | **17 (53.1%)** |  |
| I | 14 (60.9%) | 5 (71.4%) | 10 (58.8%) |  |
| II | 9 (39.1%) | 2 (28.6%) | 7 (41.2%) |  |
| **Advanced** | **24 (51,1%)** | **22 (75.9%)** | **15 (46.9%)** |  |
| III | 14 (58.3%) | 13 (59.1%) | 10 (66.7%) |  |
| IV | 10 (41.7%) | 9 (40.9%) | 5 (33.3%) |  |
| **Histopathologic grading, n (%)** | | | |  |
| Low grade (GI and GII) | 25 (53.2%) | 15 (51.7%) | 19 (59.4%) |  |
| High grade (GIII) | 22 (46.8%) | 14 (48.3%) | 13 (40.6%) |  |
| **Kurman and Shih’s type, n (%)** | | | |  |
| I | 32 (68.1%) | 18 (62.1%) | 21 (65.6%) |  |
| II | 15 (31.9%) | 11 (37.9%) | 11 (34.4%) |  |
| **Histologic type, n (%)** ^a^ | | | |  |
| Serous cystadenocarcinoma | 19 (40.4%) | 14 (48.3%) | 10 (31.3%) |  |
| Endometrioid cystadenocarcinoma | 19 (40.4%) | 8 (27.6%) | 16 (50.0%) |  |
| Mucinous cystadenocarcinoma | 6 (12.8%) | 3 (10.3%) | 2 (6.6%) |  |
| Undifferentiated carcinoma | 2 (4.3%) | 4 (13.8%) | 3 (9.4%) |  |

Abbreviations: FIGO - The International Federation of Gynecology and Obstetrics; HD – healthy donors; EOC – epithelial ovarian cancer; PB – peripheral blood; PF – peritoneal fluid; TT – tumor tissue; missing data: a n=1 (PB and TT).
